# Revealing the role of the gut microbiota in enhancing targeted therapy efficacy for lung adenocarcinoma

**DOI:** 10.1186/s40164-024-00478-7

**Published:** 2024-02-09

**Authors:** Ting Jiang, Meng Zhang, Shaoyu Hao, Shi Huang, Xin Zheng, Zheng Sun

**Affiliations:** 1 Department of Scientific Research, Qingdao Municipal Hospital of Traditional Chinese Medicine (Qingdao Hiser Medical Group), Qingdao, China; 2 Key Laboratory of Dairy Biotechnology and Engineering, Ministry of Education, Inner Mongolia Agricultural University, Hohhot, China; 3 Shandong Cancer Hospital and Institute, Shandong First Medical University and Shandong Academy of Medical Sciences, Jinan, China; 4 Faculty of Dentistry, The University of Hong Kong, Hong Kong, SAR China; 5 Channing Division of Network Medicine, Brigham and Women’s Hospital and Harvard Medical School, Boston, USA

**Keywords:** Lung adenocarcinoma, Gut microbiota, Mediation Analysis, Targeted therapy, Gefitinib efficacy

## Abstract

Lung adenocarcinoma (LUAD) is the leading cause of cancer-related death globally. Although the gut microbiota's role in the antitumor efficacy of many cancers has been revealed, its involvement in the response to gefitinib therapy for LUAD remains unclear. To fill this gap, we conducted a longitudinal study that profiled gut microbiota changes in PC-9 tumor-bearing mice under different treatments, including gefitinib monotherapy and combination therapies with probiotics, antibiotics, or Traditional Chinese Medicine (TCM). Our findings demonstrated that combining probiotics or TCM with gefitinib therapy outperformed gefitinib monotherapy, as evidenced by tumor volume, body weight, and tumor marker tests. By contrast, antibiotic intervention suppressed the antitumor efficacy of gefitinib. Notably, the temporal changes in gut microbiota were strongly correlated with the different treatments, prompting us to investigate whether there is a causal relationship between gut microbiota and the antitumor efficacy of gefitinib using Mediation Analysis (MA). Finally, our research revealed that thirteen mediators (Amplicon Sequence Variants, ASVs) regulate the antitumor effect of gefitinib, regardless of treatment. Our study provides robust evidence supporting the gut microbiota's significant and potentially causal role in mediating gefitinib treatment efficacy in mice. Our findings shed light on a novel strategy for antitumor drug development by targeting the gut microbiota.

**Supplementary Information:**

The online version contains supplementary material available at 10.1186/s40164-024-00478-7.

**To the editor,**

Lung cancer is the second most common cancer and the leading cause of death among oncologic patients worldwide, with lung adenocarcinoma (LUAD) as the predominant subtype, which accounts for approximately 40–55% of all cases [1, 2]. Despite the significant clinical benefits shown by Epidermal Growth Factor Receptor-Tyrosine Kinase Inhibitors (EGFR-TKIs) treatment in LUAD patients, the efficacy of TKIs is constrained by the widespread treatment resistance and side effects [3, 4], underscoring the urgency for developing new strategies to enhance the efficacy of EGFR-TKIs. In this regard, the gut microbiota has been illustrated to play essential roles in cancer development and the efficacy of therapeutic response [5, 6], e.g., in radiotherapy, chemotherapy, and immunotherapy for various cancers [7, 8]. Notably, diarrhea was reported as the most common side effect caused by EGFR-TKIs. Thus, we hypothesized that combination therapy with TCM or probiotics (Methods in Additional file 1) could potentially enhance the antitumor efficacy of gefitinib by modulating the host gut microbiota, while antibiotic administration will inhibit its efficacy.

## Evaluation of the antitumor effect of gefitinib alone and in combination therapies using tumor-bearing mice

**Fig. 1:**
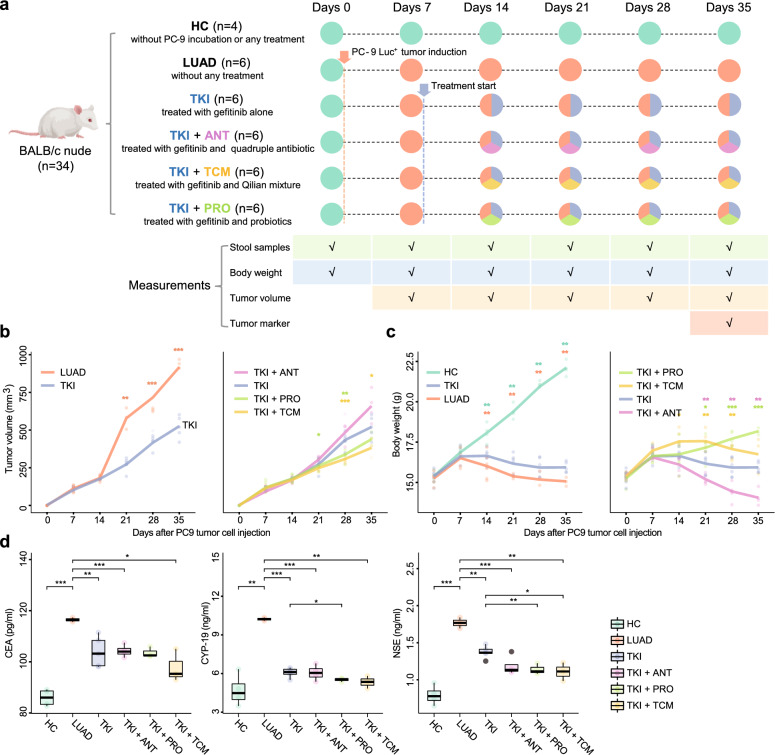
Experimental design and comparison of antitumor performance across different treatments. **a** A total of 34 mice were divided into six groups. Specifically, in addition to the healthy controls (HC, mice without PC-9 incubation or any treatment, n = 4), blank controls (LUAD, tumor-bearing mice without any treatment, n = 6), and gefitinib treatment alone (TKI, n = 6), we administered three combinational therapies, e.g., gefitinib plus antibiotic (TKI + ANT, n = 6), gefitinib plus probiotic (TKI + PRO, n = 6), and gefitinib plus Traditional Chinese Medicine (TKI + TCM, n = 6), separately to tumor-bearing mice on day 7. At baseline (day 0), approximately 5 × 10.^6^ PC9 cells were subcutaneously inoculated into the right flanks of mice except for the healthy controls (HC). Daily oral gavage of gefitinib and combinational therapies were administrated 7 days after tumor inoculation when the diameter of the tumor reached about 3 mm. Stool samples, tumor volumes, and body weights were collected or measured every 7 days from the baseline (day 0) to the end of the trial (day 35). Different colors refer to different combination treatments, e.g., pink for antibiotics, yellow for TCM, and green for probiotics. **b** The trajectories of tumor volume in different groups. In the left panel, the development of tumors in the LUAD group is compared with the TKI group, while the right panel illustrates the comparison of tumor volume between combinational therapies (TKI + ANT, TKI + PRO, TKI + TCM) and TKI at each time point. In particular, compared to TKI, the development of the tumor volume in the TKI + PRO and TKI + TCM groups was suppressed from day 21 to day 35. Although no treatment could maintain a comparable body weight development to the healthy controls, the mice in the TKI + PRO and TKI + TCM groups did not lose weight, while the body weight decreased in the TKI group and (even more dramatically) in the TKI + ANT group; **c** The trajectories of body weight in different groups. The fitting line for each group was generated using the LOWESS (Locally Weighted Scatterplot Smoothing) method. **d** The boxplots illustrate the comparison of tumor marker test results (CEA, CYP-19, and NSE) among different groups at the end of the trial. Significance levels: p-value < 0.05 (*), < 0.01 (**), < 0.001 (***), *NS* non-significance

To investigate whether the gut microbiota can alter the antitumor effect of gefitinib, we altered the microbial composition through three methods and evaluated tumor growth and progression in PC-9 Luc^+^ tumor-bearing mice (all female, n = 34, Fig. 1a). During the trial, we collected stool samples and weighed the mice every 7 days from baseline (day 0, before PC-9 induction) to the end of the trial (day 35), generating a longitudinal cohort with 204 available stool samples to study the trajectory of gut microbiota along with the progression of the tumor (Additional file 1: Fig. S1). The tumor volumes were also recorded from day 7 to day 35. After gefitinib treatment, the tumor-bearing mice exhibited reduced tumor progression (Fig. 1b). An increased antitumor effect was observed in the TKI + PRO and TKI + TCM groups (Fig. 1b, c, right panel), while a reduced antitumor effect was observed in the TKI + ANT group. To further validate the enhanced treatment efficacy, tumor markers, such as carcinoembryonic antigen (CEA), aromatase (CYP-19), and neuron-specific enolase (NSE) were tested at the end of the experiment (day 35, Fig. 1d). We found that the TKI group had significantly higher CYP-19 and NSE levels than the TKI + PRO (Student’s t-test, p = 0.027 for CYP-19 and p = 0.0097 for NSE) and TKI + TCM groups (p = 0.044 for NSE), suggesting a better antitumor efficacy in these two groups, which is in line with the observation by the tumor volume and body weight. In addition, the pathological morphology (as well as the expression of KI-67 and Caspase-3) is also in line with the tumor markers (Additional file 1: Fig. S2), suggesting an outperformed TCM and probiotic combined TKI treatment efficacy over TKI monotherapy. Notably, using TCM alone demonstrated limited independent antitumor effects (Additional file 1: Fig. S3a, b), and the in vitro cell proliferation assay (Additional file 1: Fig. S3c) revealed no significant improve the anti-tumor impact of TKI even when combining TKI with TCM. This indicates that superior antitumor results can only be attained through combination treatment in vivo, highlighting the crucial role of the gut microbiome.

## Association between the gut microbiota and different treatments

**Fig. 2:**
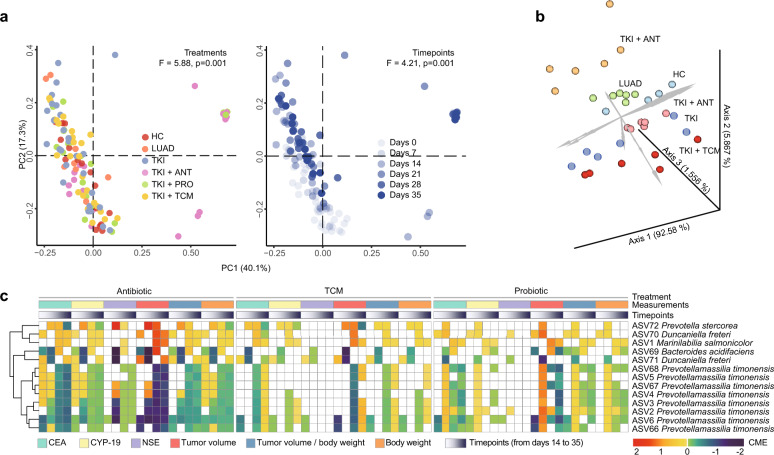
Association and causal relationship between the gut microbiota and antitumor effect of gefitinib treatments. **a** PCoA plot for all stool samples from different groups (left panel) and time points (right panel). **b** CTF based on stool microbial profiling results for all individuals from different groups. In the center of the 3D scatter plot, the healthy controls (group HC) and tumor-bearing mice without any treatment (group LUAD) were close to each other, and TKIs treatment combined with probiotics (group TKI + PRO), antibiotics (group TKI + ANT), and TCM (group TKI + TCM) were separately clustered above or below the healthy controls. **c** CME test results using antibiotics, probiotics, and TCM as treatments and different antitumor measurements as outcomes. To reduce the false-positive identification of mediators, we selected ASVs that had significant CME (with P-value < 0.05) (1) at more than two time points; (2) in more than 50% of measurements; and (3) using all three methods (antibiotic, TCM, and probiotic) as the treatment

To explore the association between different treatments (or their efficacy) and the gut microbiota, we first investigated the correlation between the antitumor measurements and the gut microbiota at each sampling time point and then analyzed the trajectory of the gut microbial composition in different groups during the trial (from day 0 to day 35) (Methods). We found that 66 ASVs from 19 genera were significantly associated with tumor progression (Spearman test P-value less than 0.05; Additional file 1: Fig. S4a, b). Moreover, the PERMANOVA Test and Principal Coordinate Analysis (PCoA, Fig. 2a) revealed a significant effect of time points (F = 4.21) and antitumor treatments (F = 5.88) on the gut microbiota. In addition, to avoid the impact of time points on microbiota data analysis, we employed Compositional Tensor Factorization (CTF) [9] to visualize the gut microbial composition of different groups (Fig. 2b). CTF reveals a clear boundary between clusters corresponding to different treatment groups, indicating that the development of the gut microbiota was profoundly changed due to the different treatments.

## A potentially causal role of the gut microbiota in enhancing the efficacy of gefitinib

The strong association between the gut microbiota and therapy efficacy encouraged us to further investigate the role of the gut microbiota (e.g., the causal role) in the varied antitumor efficacy. We employed the Sparse Microbial Causal Mediation Model (SparseMCMM [10]) to explore whether the antibiotic, probiotic, or TCM would alter gut microbiota and whether such a shift could change the antitumor efficacy of gefitinib treatment (Methods). For each combinational treatment (antibiotic, probiotic, or TCM), the OME (overall mediation effect) test revealed the significance of the gut microbiota in regulating body weight, tumor volume, and tumor markers from day 21 to 35 (Additional file 1: Table S1). This suggests that the improved (or reduced) antitumor efficacy is mediated by the gut microbiota when probiotics and TCM (or antibiotics) are used as combinational therapies in the gefitinib treatment of tumor-bearing mice. We then used the CME test (component-wise mediation effect) to determine which taxa had a significant mediation effect in enhancing or weakening the antitumor efficacy of gefitinib. We identified 13 ASVs (eight ASVs taxonomically annotated as *Prevotellamassilia* at the genus level, two *Duncaniella* ASVs, and three ASVs from *Prevotella*, *Marinilabilia*, and *Bacteroides*) that play a mediating role in the antitumor efficacy of gefitinib (Fig. 2c).

In summary, our study preliminarily revealed a causal role of the gut microbiota in modulating the anticancer efficacy of gefitinib. However, multi-omics data and a bigger sample size are warranted to provide a comprehensive and in-depth understanding of this mechanism. In our future work, to understand the mechanism behind the enhanced antitumor effect, we will explore potential metabolic interactions (such as short-chain fatty acids), immune system modifications (such as immunity and DNA damage), and alterations in the tumor microenvironment (such as lung tissue homeostasis) instigated by the gut microbiota that might influence the drug's therapeutic outcomes. Moreover, we also plan to explore the active ingredients in TCM that can regulate the gut microbiota [11, 12] and provide new pathways for enhancing TKI cancer therapy.

### Supplementary Information

**Additional file 1. **Additional Methods; Additional Figures, **Figures S1-S4**; Additional Tables, **Tables S1-S2**.

## Data Availability

The 16S rRNA sequencing data supporting this study's findings are openly available in the NCBI SRA under the project number PRJNA879426.

## Abbreviations

ANT: Antibiotics
ASVs: Amplicon Sequence Variants
CEA: Carcinoembryonic antigen
CME: Component-wise mediation effect
CTF: Compositional Tensor Factorization
CYP-19: Aromatase
EGFR-TKIs: Epidermal Growth Factor Receptor-Tyrosine Kinase Inhibitors
LUAD: Lung adenocarcinoma
MA: Mediation Analysis
ME: Mediation effect
NSE: Neuron-specific enolase
OME: Overall mediation effect
PCoA: Principal Coordinate Analysis
PRO: Probiotics
TCM: Traditional Chinese Medicine
