# Supplementary material for: Revealing the role of the gut microbiota in enhancing targeted therapy efficacy for lung adenocarcinoma
Source: Exp Hematol Oncol. 2024 Feb 9;13:15. doi: 10.1186/s40164-024-00478-7 (PMC10854116; doi:10.1186/s40164-024-00478-7)
Supplement: Supplementary file 1 — Additional file 1. Additional Methods; Additional Figures, Figures S1-S4; Additional Tables, Tables S1-S2. [file 40164_2024_478_MOESM1_ESM.pdf]

Supplementary material for

Revealing the Role of the Gut Microbiota in Enhancing Targeted Therapy Efficacy for Lung adenocarcinoma

Supplemental Methods

Supplemental Figures

Figure S1. Data collection scheme for stool samples and subject information.

Figure S2. HE staining of tumor tissues and expression of KI-67 and Caspase-3 in each group at the end of the trial (day 35).

Figure S3. Comparison of antitumor performance across different treatments.

Figure S4. Identification of associated ASVs with antitumor effect of gefitinib.

Supplemental Tables

Table S1. OME test results using antibiotic, TCM, and probiotic as treatments and different antitumor measurements as outcome.

Table S2. Ingredients of the Traditional Chinese Medicine used in this study.

Reference

Supplemental methods

Additives and drugs gefitinib (250 mg film-coated tablets) were provided by CHIA TAI TIANQING (CTTQ) Pharmaceutical Co., Ltd. (Nanjing, China). We obtained quadruple antibiotic[1](containing 0.5 g/L vancomycin, 1 g/L neomycin, 1 g/L metronidazole, and 1 g/L ampicillin) from Qingdao Jieshikang Biotechnology Co., Ltd. (Qingdao, China). Probiotics (product number:108.880302) were purchased from Baoying Co., Ltd. (Beijing, China), which contained *Lactobacillus acidophilus* 2.0×10^{11} cfu and *Bifidobacterium lactis* 0.2×10^{11} cfu per 100 g). The formula for the Qilian mixture is listed in **Table S2**. All the herbs were provided by NEAUTUS Co., Ltd. (Chengdu, China). The mixture was prepared at the Class III Laboratory of Traditional Chinese Medicine, Qingdao Hospital of Traditional Chinese Medicine.

Cell line preparation The PC-9 cell line (an EGFR-mutated human lung cancer cell line) was provided by the Chinese Academy of Sciences Cell Bank (Shanghai, China). These cell lines were cultured in RPMI-1640 medium (Meilunbio, China) supplemented with 10% fetal bovine serum (FBS; Meilunbio, China) and antibiotics (100 u/ml penicillin and 100 μg/ml streptomycin; Meilunbio, China) in a humidified 5% CO_2 incubator at 37 °C [2] before use.

Cell proliferation assay We conducted the Cell Counting Kit-8 (CCK-8) assays (Meilunbio, China) to assess the impact of the TCM (Qilian mixture) and the gefitinib, both individually and in combination, on the proliferation of PC9 cells. We seeded the cells in a 96-well plate and treated the PC9 cells with either the TCM alone, gefitinib alone, or gefitinib and TCM for 48 hours. Subsequently, we added the CCK-8 reagent and incubated it at 37°C for 2 hours. The absorbance at 450 nm was then measured using the Victor Nivo microplate reader (PerkinElmer, USA) to calculate relative cell vitality.

Mice Four-week-old BALB/c nude mice (18g-20g, n=34, all female) were purchased from Huafukang Laboratory Animal Co., Ltd. (China, license number: SCXK (Beijing) 2020-0004). Nude mice were housed in the SPF animal room of the animal platform at Qingdao University. The experimental animals were licensed under SYXK (Lu) 2020 0009. The mice were housed under pathogen-free conditions in groups with access to sterile water and food. Temperatures indoors range between 22°C and 25°C, humidity is 60%, and there is a 12-hour light and dark cycle.

Experimental design and model construction A total of 34 mice were divided into six groups: healthy control (HC, mice without PC-9 incubation or any treatment, n=4), blank control (LUAD, tumor-bearing mice without any treatment, n=6), TKI (tumor-bearing mice treated with gefitinib alone, 32.5 mg/kg per day, n=6), TKI+TCM (tumor-bearing mice treated with gefitinib 32.5 mg/kg per day and Qilian mixture 10.4mL/kg $12h^{-1}$, n=6), TKI+ANT (tumor-bearing mice treated with gefitinib 32.5 mg/kg per day and quadruple antibiotic 10 ml/kg $12h^{-1}$, n=6), and TKIs + PRO (tumor-bearing mice treated with gefitinib 32.5 mg/kg per day and probiotic 0.2 g/kg $12h^{-1}$, n=6). At baseline, approximately 5×10^6 PC9 cells were subcutaneously inoculated into the right flank of mice, except for healthy controls (HC). Daily oral gavage gefitinib and combination therapies were administrated seven days after tumor inoculation when the diameter of the tumor reached approximately 3 mm. Body weight and stool samples were measured and collected every seven days from baseline (day 0) to the end of the trial (day 35). The tumor volume (V, mm^3) was measured every seven days from day 7 to day 35, e.g., we measured the longest diameter (L, mm) and its vertical width (W, mm) of the tumor with a vernier caliper, and tumor volume was calculated by $V = (L \times W^2)/2$. In addition to the mice mentioned above, we also included 6 mice with TCM alone (Qilian mixture 10.4mL/kg $12h^{-1}$) as treatment for comparison.

Pathomorphological observation After sacrificing the mice, the tumor body was taken, and the central necrotic tissue and surrounding connective tissue were stripped and fixed with 4% paraformaldehyde. Then, the tumor tissue was embedded in paraffin, and the pathological status was observed after hematoxylin-eosin (HE) staining.

Detection of KI-67 and Caspase-3 We first fixed the tissue specimens with 4% paraformaldehyde, followed by routine paraffin embedding, and then prepared tissue sections with a thickness of 4μm. After baking and deparaffinizing the tissue sections, we boiled them in an antigen retrieval solution with a pH of 6. The sections were then blocked using a solution containing 5% BSA and 1% goat serum and incubated overnight at 4°C with primary antibodies against KI-67 or Caspase-3. Subsequently, we incubated the tissue sections according to the instructions provided with the VECTASTAIN® Elite® ABC HRP Kit (PK-6100, VECTOR, USA) and ImmPACT® DAB Peroxidase (HRP) Substrate (SK-4105, VECTOR, USA). The stained tissue sections were then photographed under a microscope (×200 magnification).

Serum tumor marker test (ELISA) At the end of the trial (day 35, after sample collection and measurement), the mice in each group were anesthetized, and their blood was collected. Blood samples were left standing for 30 minutes at 3000 rpm for 10 minutes, and the supernatant (serum) was then stored in the - 80 ℃ refrigerator. The serum levels of tumor markers (NSE, CEA, and CYP-19) were detected using the Mouse Neuron-specific enolase (NSE) ELISA Kit (BS-E9103M2 48T, JSBOSSEN, China), mouse carcinoembryonic antigen (CEA) ELISA Kit (BS-E8644M2 48T, JSBOSSEN, China), and mouse cytochrome P450-19 (CYP-19) ELISA Kit (BS-E19403M2 48T, JSBOSSEN, China), respectively, and analyzed according to the manufacturer's instructions. We then used an enzyme labeling analyzer (Thermo Fisher, US) to display the absorbance of NSE, CEA, and CYP-19 at 450 nm and used a standard curve to calculate their concentrations in the samples.

Fecal DNA extraction and 16S rRNA sequencing At days 0, 7, 14, 21, 28, and 35, fresh fecal samples of mice were directly collected from the anus, flash-frozen in liquid nitrogen for 1 min, and then transferred to a-80°C refrigerator. V3-V4 region (primers 341F/785R were used, and the target insert length was approximately 450bp) of 16S rDNA was amplified using the KAPA HiFi Hotstart PCR Kit (KAPA Biosystems) based on the manufacturer’s protocol (KAPA Biosystems) and approximately 50 ng of extracted DNA per reaction. The thermocycling conditions were set at 95°C for 1 min, 55°C for 1 min, and 72°C for 1 min for 30 cycles, followed by a final extension at 72°C for 5 min. PCR reactions were performed in 50 ml triplicate and then combined. The MiniElute® Gel Extraction Kit (QIAGEN) was used to extract PCR products, which were then quantified with a NanoDrop ND-1000 spectrophotometer and a Qubit 2.0 Fluorometer (Thermo Electron Corporation). After purification, the amplicons were pooled in equimolar concentrations and their final concentration was determined using Qubit (Invitrogen).

16S rRNA sequencing data analysis Raw sequencing reads were demultiplexed and quality-filtered using the standard procedures of QIIME 2 (Bolyen et al., 2019, version 2021.4) with default parameters [3, 4]. The DADA2 (Callahan et al., 2016) plugin (version 2021.4.0) of QIIME2[5] was used to denoise, de-replicate, and count amplicon sequence variants (ASVs), incorporating the following parameters: (1) forward and reverse reads were truncated to 150 bases; (2) forward and reverse reads with the number of expected errors higher than 2.0 were

discarded; (3) chimeras were detected using the "consensus" method and removed. Taxonomies were assigned to the final sequences using the Silva database (v138-99) and classify-sklearn procedure. Chloroplastic and mitochondrial ASVs were removed. For other statistical analyses of the gut microbiota, Parallel-Meta 3.5 [6] was used to calculate and demonstrate beta diversity (PCoA) based on the ASVs. Differences in beta diversity based on Bray-Curtis dissimilarity were determined using non-parametric multivariate analysis of variance (PERMANOVA) with 999 random permutations. Data visualization in this study was performed using R (v4.0.2), e.g., the "ggplot2," "ggpubr" package were used for boxplot, scatter plot, and line chart, and the "pheatmap" was employed for the heatmaps.

To clearly illustrate the differences between treatments instead of time points, we visualized the differences in microbial composition between different treatments using Context-aware Tensor Factorization (CTF)[3, 7]. CTF is explicitly designed for metagenomic sequencing data and substantially utilizes the compositionality and sparsity of the data by finding a CP decomposition that best approximates non-zero values.

Mediation analysis SparseMCMM is a high-dimensional and compositional aware method designed to estimate the causal mediation effect (ME) of microbiota using three factors (treatment, microbiome, and outcome). In particular, SparseMCMM used linear log-contrast regression and Dirichlet regression to model the causal mediation relationships of treatment, microbiome, covariates, and outcomes and provided a clear and sensible causal path among treatment, microbial composition, and outcome under four sufficient identifiable assumptions. Here, we examined whether the overall ME of the microbiome community on the outcome was significant (OME test) or whether at least one component-wise ME was significantly non-zero (CME test). Specifically, we assigned the three factors required by the SparseMCMM: (1) we leveraged the samples from gefitinib alone (TKI) and any one of the combinational therapies (TKI+ANT, TKI+PRO, or TKI+TCM) to set the antibiotic, probiotic, or TCM as the treatment; (2) we used the longitudinal 16S rRNA data as the microbiome; and (3) we considered the antitumor measurements at the end of the trial (day 35) as the outcome. Significant results were derived at the ASV level, and only ASVs with a relative abundance higher than 0.1% and a prevalence higher than 30% were retained. P-values were obtained with 1000 permutations.

Supplemental Figures

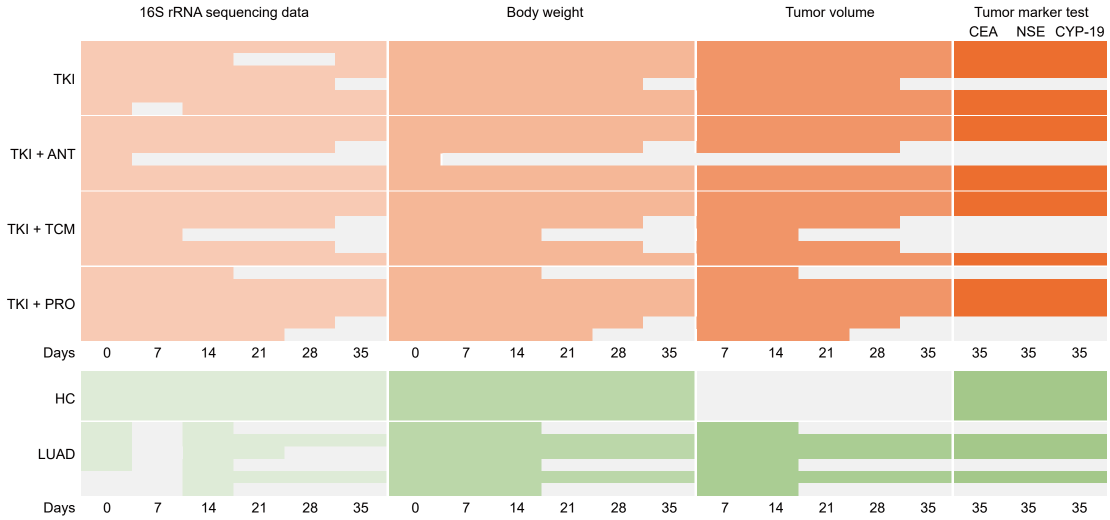

Figure S1. Data collection scheme for stool samples and subject information. Rows represent individuals, and columns represent time points. Grey blocks refer to missing collection or failure in sequencing. A total of 34 mice were divided into six groups: Healthy control (HC, mice without PC-9 incubation or any treatment, 4 sequenced stool samples), Blank control (LUAD, tumor-bearing mice without any treatment, 6 sequenced stool samples), TKI (tumor-bearing mice treated with gefitinib alone, 6 sequenced stool samples), TKI+TCM (tumor-bearing mice treated with gefitinib and Qilian mixture, 6 sequenced stool samples), TKI+ANT (tumor-bearing mice treated with gefitinib and quadruple antibiotic, 6 sequenced stool samples), and TKIs + PRO (tumor-bearing mice treated with gefitinib and probiotic, 6 sequenced stool samples). Subject information (e.g., body weight, tumor volume, and marker test results) for most of the sequenced stool samples were collected.

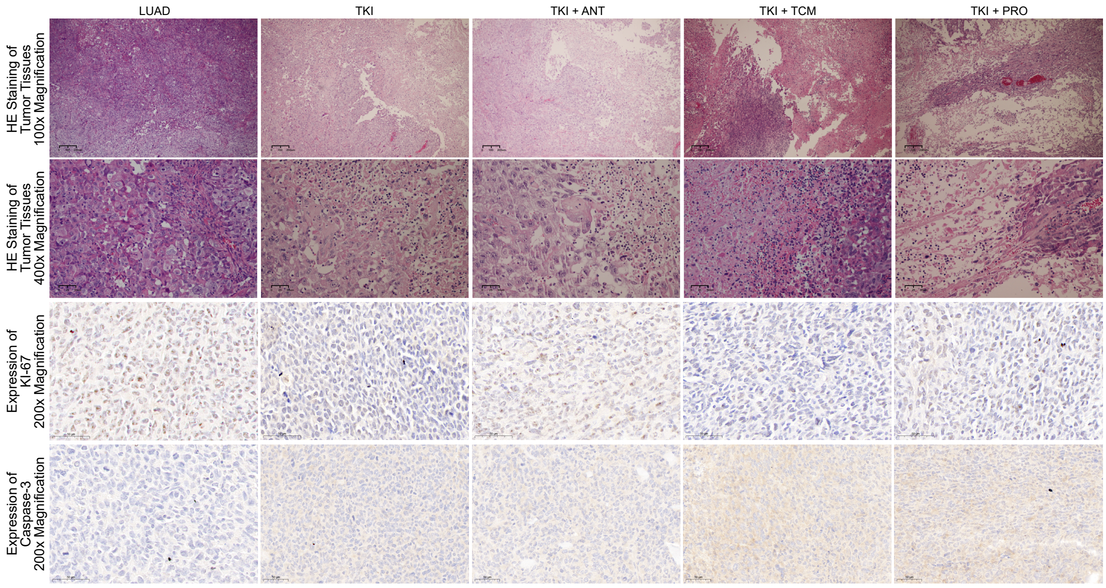

Figure S2. HE staining of tumor tissues and expression KI-67 and Caspase-3 in each group at the end of the trial (day 35). The tumor cells were observed in all groups with diffuse and lamellar arrangement, slender fibrovascular interstitium, strong intercellular adhesion, round or round-like cells, eosinophilic cytoplasm, 1-2 centered nucleoli, and easy-to-see nuclear fission images, which proved the success of PC-9 tumor-bearing mouse model. Moreover, we found that the tumor cell necrosis was about 5% in the LUAD group with mild inflammatory cell reaction, 50% in the TKI group with moderate inflammatory cell reaction, 35% in the TK+ANT group with moderate inflammatory cell reaction, 75% in the TKI+TCM group with severe inflammatory cell reaction, and the tumor cell necrosis was about 75% in the TKI+TCM group with severe inflammatory cell reaction. In the TKI+PRO group, about 75% of tumor cell necrosis was seen with severe inflammatory response. At bottom, tumor tissues were subjected to immunohistochemical analysis of KI-67 and Caspase-3 (scale bar, 50 μm). The combination of the TCM and gefitinib resulted in decreased KI-67 expression and increased Caspase-3 expression.

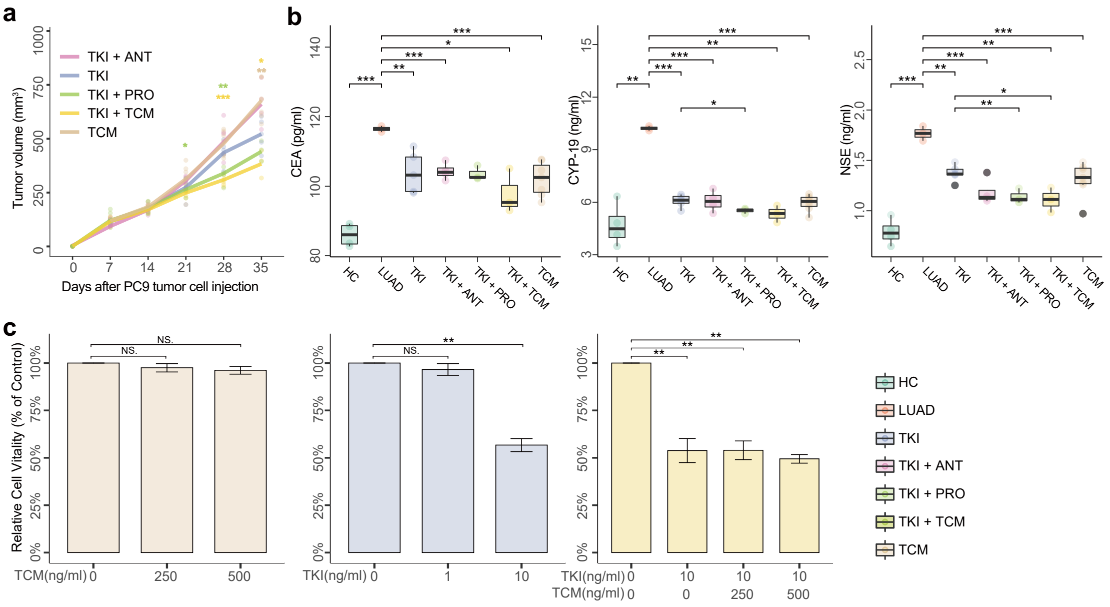

Figure S3. Comparison of antitumor performance across different treatments (**a**) The trajectories of tumor volume in different groups (TKI, TKI + ANT, TKI + PRO, TKI + TCM, and TCM alone). (**b**) The boxplots illustrate the comparison of tumor marker test results (CEA, CYP-19, and NSE) among different groups at the end of the trial. (**c**) The effect of the Qilian Mixture and gefitinib (both alone and in combination) on PC9 cell proliferation (in vitro). The left and middle panels display the proliferation of PC9 cells when treated with either the Qilian Mixture (0, 250, 500 ng/ml) alone or gefitinib (0, 1, 10 nmol/L) alone. The right panel showcases the proliferation of PC9 cells treated with a combination of the Qilian Mixture (0, 250, 500 ng/ml) and gefitinib (0 or 10 nmol/L).

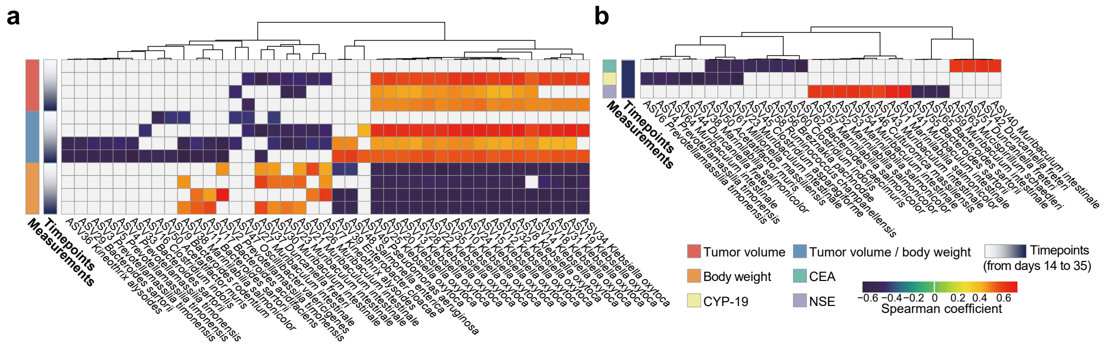

Figure S4. Identification of associated ASVs with antitumor effect of gefitinib. (**a**) The heat map shows the Spearman correlation between antitumor measurements (e.g., tumor volume, normalized tumor volume, and body weight, measured from day 14 to 35) and the specific gut microbiota. (**b**) The correlation between tumor marker test results and the gut microbiota at day 35. In particular, 19 ASVs taxonomically annotated as *Enterobacter*, *Klebsiella*, *Pseudomonas*, and *Salmonella* were found to be positively correlated with tumor volume and negatively correlated with body weight, whereas 22 ASVs belonging to *Acetatifactor*, *Bacteroides*, *Clostridium*, *Duncaniella*, *Kineothrix*, *Marinilabilia*, *Muribaculum*, *Oscillibacter*, *and Prevotellamassilia* were negatively correlated with tumor volume and positively correlated with body weight. In addition to tumor volume and body weight, we calculated the correlations between the tumor marker test results and gut microbiota at the end of the trial (day 35). Specifically, we identified 12 ASVs annotated as *Marinilabilia*, *Muribaculum*, *Duncaniella*, *Culturomica*, and *Mucispirillum* that were negatively associated with CEA, CYP-19, and NSE, while 16 ASVs from *Marinilabilia*, *Prevotellamassilia*, *Duncaniella*, *Clostridium*, *Acetatifactor*, *Bacteroides*, *Breznakia*, *Ruminococcus*, *Muribaculum*, and *Millionella* were positively associated with CEA and NSE.

Supplemental Tables

Measurements	Treatment	Day 14	Day 21	Day 28	Day 35
**Body weight**	Antibiotics	1.328	1.121	1.289	1.236
	TCM	0.264	0.595	0.835	0.357
	Probiotics	0.360	0.812	0.715	0.587
**Tumor volume**	Antibiotics	2.977	2.817	2.985	2.957
	TCM	-1.589	-0.211	1.820	-1.593
	Probiotics	-1.493	1.531	1.459	0.938
**Tumor volume / body weight**	Antibiotics	1.836	1.686	1.852	1.825
	TCM	-0.697	-0.265	0.631	-0.610
	Probiotics	-0.661	-0.399	-0.265	-0.390
**CEA**	Antibiotics	2.177	1.121	2.160	2.104
	TCM	-0.381	1.077	1.486	0.353
	Probiotics	0.307	1.323	1.245	1.053
**NSE**	Antibiotics	0.441	2.817	0.383	0.373
	TCM	-0.135	0.022	0.082	-0.024
	Probiotics	-0.044	0.006	0.009	0.004
**CYP_19**	Antibiotics	0.990	1.686	0.958	0.905
	TCM	-0.170	0.150	0.367	-0.045
	Probiotics	-0.087	0.207	0.174	0.103

Table S1. OME test results using antibiotic, TCM, and probiotic as treatments and different antitumor measurements as outcome. Significant overall mediation effect of the gut microbiota on antitumor effect of gefitinib is highlighted with green.

Herb name	Family	Part used	Amount used (g)
Radix Astragali (Astragalus membranaceus (Fisch.) Bunge.)	Leguminous	Root	87
Radix ginseng (Panax ginseng C. A. Meyer.)	Araliaceae	Root	51
Rhizoma Atractylodis macrocephalae (Atractylodes macrocephala Koidz.)	Asteraceae Bercht. & J. Presl	rhizom	45
Poria cocos (Wolfiporia cocos (F.A. Wolf) Ryvarden & Gilb.)	Polyporaceae	sclerotium	45
Herba Scutellariae barbatae (Scutellaria barbata D. Don)	Labiatae	grass	87
Herba Hedyotis Diffusae (Hedyotis diffusa Willd.)	Rubiaceae	grass	87
Bulbus Fritillariae thunbergii (Fritillaria thunbergii Miq.)	Liliaceae	bulbus	67
Pseudobulbus Cremastrae seu Pleiones (Asarum sagittarioides C. F. Liang)	Orchid	bulbus	33
semen coicis (Coix lacryma-jobi)	Gramineae	seed	87
Radix Glycyrrhizae (Glycyrrhiza uralensis Fisch.)	Leguminosae	Root	33

Table S2. Ingredients of the Traditional Chinese Medicine used in this study.

Preparation process: (1) To the above ten herbs, add 10 times the amount of water, let them soak for 2 h, cook for 2 h, and then filter the rest out; (2) Add 10 times more water, cook for 2 h, then strain; (3) The two filtrates (from 1 and 2) were combined and concentrated into a clear paste with a relative density of 1.06~1.10 (60 °C) under reduced pressure; (4) The paste was filtered (120 mesh) after standing for 24 h, and then 500mL of water was added and mixed, and then divided into 250mL sodium-calcium glass infusion bottles, sealed, and sterilized under hot pressure (115 °C) for 50 min.

Reference

1. Chadchan SB, Cheng M, Parnell LA, Yin Y, Schriefer A, Mysorekar IU, Kommagani R: **Antibiotic therapy with metronidazole reduces endometriosis disease progression in mice: a potential role for gut microbiota**. *Hum Reprod* 2019, **34**(6):1106-1116.
2. Jarry U, Bostoen M, Pineau R, Chaillot L, Mennessier V, Montagne P, Motte E, Gournay M, Le Goff A, Guillaudeux T *et al*: **Correction to: Orthotopic model of lung cancer: isolation of bone micro-metastases after tumor escape from Osimertinib treatment**. *BMC Cancer* 2021, **21**(1):574.
3. Hendrickson R, Urbaniak C, Minich JJ, Aronson HS, Martino C, Stepanauskas R, Knight R, Venkateswaran K: **Clean room microbiome complexity impacts planetary protection bioburden**. *Microbiome* 2021, **9**(1).
4. Bolyen E, Rideout JR, Dillon MR, Bokulich N, Abnet CC, Al-Ghalith GA, Alexander H, Alm EJ, Arumugam M, Asnicar F *et al*: **Reproducible, interactive, scalable and extensible microbiome data science using QIIME 2**. *Nature biotechnology* 2019, **37**(8):852-857.
5. Russell A, Copio JN, Shi YS, Kang SM, Franklin CL, Ericsson AC: **Reduced housing density improves statistical power of murine gut microbiota studies**. *Cell Rep* 2022, **39**(6).
6. Jing GC, Sun Z, Wang HL, Gong YH, Huang S, Ning K, Xu J, Su XQ: **Parallel-META 3: Comprehensive taxonomical and functional analysis platform for efficient comparison of microbial communities**. *Scientific reports* 2017, **7**:40371.
7. Mor U, Cohen Y, Valdés-Mas R, Kviatcovsky D, Elinav E, Avron H: **Dimensionality reduction of longitudinal 'omics data using modern tensor factorizations**. *Plos Comput Biol* 2022, **18**(7):e1010212.
